# Intensive prolonged exposure therapy for chronic PTSD patients following multiple trauma and multiple treatment attempts

**DOI:** 10.1080/20008198.2018.1425574

**Published:** 2018-01-30

**Authors:** Lotte Hendriks, Rianne A. de Kleine, Theo G. Broekman, Gert-Jan Hendriks, Agnes van Minnen

**Affiliations:** ^a^ Overwaal Centre of Expertise for Anxiety Disorders, OCD and PTSD, Institution for Integrated Mental Health Care Pro Persona, Nijmegen, The Netherlands; ^b^ Behavioural Science Institute, NijCare, Radboud University, Nijmegen, The Netherlands; ^c^ Institute of Psychology, Leiden University, Leiden, The Netherlands; ^d^ Bureau Bêta, Nijmegen, The Netherlands; ^e^ Department of Psychiatry, Radboud University Medical Centre, Nijmegen, The Netherlands; ^f^ Psychotrauma Expertise Centre (PSYTREC), Bilthoven, The Netherlands

**Keywords:** (complex) PTSD, treatment outcome, (prolonged) exposure, intensive treatment, predictors, response patterns, TEPT (complejo), resultado del tratamiento, exposición (prolongada), tratamiento intensivo, predictores, patrones de respuesta, （复杂）PTSD, 治疗结果, （延长）暴露, 强化治疗, 预测指标, 反应模式, • Current trauma-focused treatment (TFT), including prolonged exposure (PE), usually lasts several months with sessions being delivered on a weekly basis.• In this study, PE is administered in an accelerated way in chronic PTSD patients with a likely diagnosis of ICD-11 Complex PTSD following multiple interpersonal trauma and a history of multiple treatment attempts.• The results of this open study suggest that intensive PE (iPE) can be effective. Although previous treatment attempts were unsuccessful in these patients, 71% showed partial or complete response during iPE. In addition, iPE was found to be safe and dropout rates were very low.

## Abstract

**Background**: Suboptimal response and high dropout rates leave room for improvement of trauma-focused treatment (TFT) effectiveness in ameliorating posttraumatic stress disorder (PTSD) symptoms.

**Objective**: To explore the effectiveness and safety of intensive prolonged exposure (iPE) targeting chronic PTSD patients with a likely diagnosis of ICD-11 Complex PTSD following multiple interpersonal trauma and a history of multiple treatment attempts.

**Method**: Participants (*N *= 73) received iPE in 12 × 90-minute sessions over four days (intensive phase) followed by four weekly 90-minute booster prolonged exposure (PE) sessions (booster phase). The primary outcomes, clinician-rated severity of PTSD symptoms, and diagnostic status (Clinician-Administered PTSD Scale; CAPS-IV) were assessed at baseline, post-treatment, and at three and six months. Treatment response trajectories were identified and predictors of these trajectories explored.

**Results**: Mixed model repeated measures analysis of CAPS-IV scores showed a baseline-to-posttreatment decrease in PTSD symptom severity (*p *< .001) that persisted during the three- and six-month follow-ups with large effect sizes (Cohen’s *d* > 1.2); 71% of the participants responded. None of the participants dropped out during the intensive phase and only 5% during the booster phase. Adverse events were extremely low and only a minority showed symptom exacerbation. Cluster analysis demonstrated four treatment response trajectories: *Fast responders* (13%), *Slow responders* (26%), *Partial responders* (32%), and *Non-responders* (29%). Living condition and between-session fear habituation were found to predict outcome. Participants living alone were more likely to belong to the *Partial responders* than to the *Non-responders* cluster, and participants showing more between-session fear habituation were more likely to belong to the *Fast responders* than to the *Non-responders* cluster.

**Conclusions**: The results of this open study suggest that iPE can be effective in PTSD patients with multiple interpersonal trauma and after multiple previous treatment attempts. In addition, in this chronic PTSD population iPE was safe.

## Abstract (es)

**Planteamiento**: La respuesta subóptima y las altas tasas de abandono dejan margen para la mejora de la eficacia del tratamiento centrado en el trauma (TCT) en la mejora de los síntomas del trastorno por estrés postraumático (TEPT).

**Objetivo**: explorar la efectividad y la seguridad de la exposición prolongada intensiva (EPI) dirigida a pacientes con TEPT crónico con un probable diagnóstico de TEPT complejo de la CIE-11 después de múltiples traumas interpersonales y un historial de múltiples intentos de tratamiento.

**Método**: Los participantes (*N* = 73) recibieron EPI en 12 sesiones de 90 minutos durante cuatro días (fase intensiva) seguidas de cuatro sesiones semanales de exposición prolongada (EP) de refuerzo de 90 minutos (fase de refuerzo). Los resultados principales, la gravedad de los síntomas del TEPT evaluados por el clínico y el estado diagnóstico evaluados por el clínico (Escala de TEPT administrada por el clínico, CAPS-IV, por sus siglas en inglés) se evaluaron al inicio, después del tratamiento, y a los tres y seis meses. Se identificaron las trayectorias de respuesta al tratamiento y se exploraron los predictores de estas trayectorias.

**Resultados**: Los análisis de medidas repetidas de las puntuaciones de CAPS-IV desde un modelo mixto mostraron una disminución de la línea de base hasta el postratamiento en cuanto a la gravedad de los síntomas de TEPT (*p* <.001) que persistió durante los seguimientos a los 3 y 6 meses con tamaños de efecto grandes (d de Cohen> 1,2); el 71% de los participantes respondieron. Ninguno de los participantes abandonó durante la fase intensiva y solo el 5% lo hizo durante la fase de refuerzo. Los eventos adversos fueron extremadamente bajos y solo una minoría mostró exacerbación de los síntomas. El análisis de clusters demostró cuatro trayectorias de respuesta al tratamiento: *los que responden rápidamente* (13%), *los que responden lentamente* (26%), *los que responden parcialmente* (32%) *y los que no responden* (29%). Se descubrió que las condiciones de vida y la habituación al miedo entre sesiones predecían el resultado. Los participantes que vivían solos eran más propensos a pertenecer a *los que responden parcialmente* que al grupo de *los que no responden*, y los participantes que demostraron más habituación al miedo entre sesiones tenían más probabilidades de pertenecer a *los que responden rápidamente* que al grupo de *los que no responden*.

**Conclusiones**: los resultados de este estudio abierto sugieren que la EPI puede ser efectiva en pacientes con TEPT con traumas interpersonales múltiples y después de múltiples intentos previos de tratamiento. Además, en esta población de TEPT crónico, la EPI era segura.

## Abstract (zh)

**背景**：效果欠佳和高脱落率说明用于减轻创伤后应激障碍症状（PTSD）的创伤中心治疗（TFT）的效果还有提升空间。

**目标**：探索用于长期 PTSD患者强化延长暴露（iPE）的效果和安全性。这些病人经历过多次人际创伤，有多次治疗尝试，有可能被同时诊断为 ICD-11复杂 PTSD。

**方法**：被试（n=73）首先在四天时间里接受12次 X90分钟的 iPE 疗程（强化阶段），随后接受四次每周进行的90分钟增进延长暴露（PE）疗程（增强阶段）。在基线期、治疗后和3月、6月后各评估一次主要结果——治疗师评估的 PTSD 症状严重度和诊断状况（临床医生用 PTSD 量表；CAPS-IV）。识别治疗反应轨迹，并探索这些轨迹分类的预测指标。

**结果**：对 CAPS-IV 分数进行重复测量混合模型分析显示 PTSD 症状严重度从基线到治疗后有所减轻（p <.001），并在3月和六月后的追踪期得到保持，症状减轻程度的效应量显著（Cohen’s *d* > 1.2）。71%的被试在治疗后有所反应。没有被试在强化阶段脱落，只有5%的被试在增强阶段脱落。负性事件非常少发生，只有 少数人出现了症状恶化。聚类分析显示四个治疗反应轨迹：快速有效（13%），缓慢有效（26%），部分有效（32%）和无效（29%）。生活质量和治疗间恐惧习惯化可以预测结果。独自居住的被试更有可能是部分有效组（相较于无效组）；出现更多的治疗间恐惧性习惯化的被试更有可能是快速有效组（相较于无效组）。

**结论**：这项开放研究的结果显示 iPE 可以有效地用于 有多次人际创伤和多次前期治疗尝试的PTSD 病人。另外这种用于长期 PTD 人群的 iPE 是安全的。

## 1. Introduction

Trauma-focused treatment (TFT) programmes have strong empirical support for their effectiveness in ameliorating posttraumatic stress disorder (PTSD) symptoms (Cusack et al., 2016). Still, a systematic review of response in anxiety related disorders showed that the average response rate for PTSD is approximately 60% (Loerinc et al., 2015), although it needs to be noted that definitions of treatment response varied greatly across studies. In addition to this suboptimal response, dropout rates in regular TFT programmes are high. A meta-analysis investigating dropout from PTSD treatment, including TFT programmes and supportive counselling (Imel, Laska, Jakupcak, & Simpson, 2013), estimated that, on average, 18% of patients drop out, with percentages varying significantly across studies and rates running up to 52%, although definitions of dropout differed among the studies.

To improve both response and dropout, TFT programmes have been augmented in various ways (de Kleine, Rothbaum, & van Minnen, 2013; Kehle-Forbes et al., 2013). However, these modifications have not resulted in clinically significant improvement of either outcome or dropout rates. A relatively new strategy is to deliver the treatment sessions in a highly intensive format (Hendriks, de Kleine, Hendriks, & van Minnen, 2016; Rauch & Rothbaum, 2016), with patients attending multiple sessions within a compact period of time (e.g. within one week) instead of weekly sessions over the course of several months. The main argument for intensifying TFT is the expectation that it will improve both outcome and dropout.

Indeed, a higher session frequency resulted in faster recovery in psychotherapy programmes in general (Erekson, Lambert, & Eggett, 2015). Regarding TFT programmes for PTSD, more specifically Cognitive Processing Therapy (CPT) or Prolonged Exposure (PE), higher session frequencies were associated with significantly greater PTSD symptom amelioration even when controlling for the amount of sessions (Gutner, Suvak, Sloan, & Resick, 2016). The authors suggested that intensifying TFT may help reduce avoidance of confronting the details of (situations related to) the trauma memories. More theoretically, results from animal studies about fear extinction learning (i.e. the development of new associations with the stimulus that inhibits the manifestation of the original fear memory and the presumed mechanism of action of exposure therapy; see for an overview Fitzgerald, Seemann, & Maren, 2014) showed that once extinction learning is initiated in a massed way, further extinction learning is more effective when trials are spaced (Cain, Blouin, & Barad, 2003).

Additionally, highly intensive treatments may improve dropout rates in the treatment of PTSD. For instance, in the meta-analysis of Imel and colleagues (2013), variability in dropout rates across studies was associated with the number of sessions delivered. That is, in treatment programmes that encompassed more (weekly) sessions, patients were more likely to drop out. This suggests that shorter treatment durations might prove superior in retaining patients. While treatment dropout is generally considered a negative outcome, a recent study found that some patients that prematurely ended CPT or PE nevertheless showed significant PTSD symptom amelioration, although having attended more treatment sessions was associated with better treatment outcomes (Szafranski, Smith, Gros, & Resick, 2017). These findings suggest that, while it is important that treatment duration is kept as short as possible to prevent dropout and improve treatment outcome, the total number of treatment sessions should not be reduced.

Although abovementioned studies suggest that a condensed TFT delivery format is a plausible strategy to improve response and reduce dropout, the effectiveness of these so-called intensive TFT programmes is still largely unknown. One study used a brief imaginal exposure therapy (five daily 50-minute sessions) to determine the augmentation effects of methylene blue versus placebo and compared outcomes to a waiting list condition that was later converted into TFT delivered twice weekly. The study provided preliminary evidence that intensive imaginal exposure was effective, with results being comparable to those obtained with regular treatment with twice weekly sessions (Zoellner et al., 2017). To our knowledge, only one randomized controlled trial (RCT) directly compared intensive TFT with TFT delivered in weekly sessions. This study by Ehlers and colleagues (2014) showed that an 18-hour intensive cognitive therapy delivered within one week showed faster symptom reduction and was equally effective in ameliorating PTSD symptoms as regular weekly cognitive therapy for patients suffering from PTSD following a single trauma in adulthood. Additionally, the dropout rate for the intensive programme was remarkably low (3%), however, it must be mentioned that the regular weekly cognitive therapy showed a comparable low dropout rate. The authors emphasized that intensive TFT is of interest when treatment needs to be conducted within a short period of time or when patients themselves indicate a preference for condensed treatment. However, the effectiveness of highly intensive TFT for more complex patient populations, such as patients with a history of multiple childhood trauma or patients meeting the symptoms of the ICD-11 diagnosis of complex PTSD as proposed by the World Health Organization (WHO; Maercker et al., 2013) that includes PTSD symptoms as well as disturbances in affect dysregulation, negative self-concept, and interpersonal problems, is still largely unknown.

There is a lot of discussion about the treatment of this so-called complex PTSD group, with some clinicians and researchers highlighting the importance of tailoring treatments to this population, for example by providing sequential or multicomponent therapies instead of stand-alone TFT (Cloitre, 2015). Unlike intensive TFT, this would imply treatments being lengthened rather than shortened. Others argue that the evidence for these sequential or multicomponent interventions for complex PTSD patients is weak (de Jongh et al., 2016). To bridge the knowledge gap concerning the effectiveness of TFT for more complex patient populations, we developed a highly intensive TFT programme for those patients with a likely diagnosis of ICD-11 Complex PTSD after multiple interpersonal trauma that had a history of multiple treatment attempts as a next step in their treatment. The intervention is offered in a massed format of 12 × 90-minute sessions during four days and is based on PE, a first line treatment for PTSD (Powers, Halpern, Ferenschak, Gillihan, & Foa, 2010) and recommended in available treatment guidelines (e.g. National Institute for Health and Clinical Excellence Guidelines on PTSD (NICE), 2005; International Society for Traumatic Stress Studies, 2009).

In standard weekly TFT programmes, patients characteristics are not found to be stable predictors of treatment outcome (Ehlers et al., 2013; Ehring et al., 2014; Powers et al., 2010; van Minnen, Arntz, & Keijsers, 2002; van Minnen, Harned, Zoellner, & Mills, 2012) and findings concerning (early) treatment process variables, such as fear habituation, are inconsistent (Bluett, Zoellner, & Feeny, 2014; Sripada & Rauch, 2015; van Minnen & Hagenaars, 2002). This variability might be explained by the fact that in most studies prediction analyses of associations were performed in entire and thus heterogeneous samples. In response to this methodology, researchers recently identified several distinct trajectories of treatment response based on change patterns, suggesting there are disparate, more homogeneous, response groups that might have differential predictors (Allan, Gros, Myers, Korte, & Acierno, 2016; Clapp, Kemp, Cox, & Tuerk, 2016; Galovski et al., 2016; Stein, Dickstein, Schuster, Litz, & Resick, 2012).

The main purpose of the present open clinical trial was to investigate whether the highly intensive prolonged exposure (iPE) programme would decrease PTSD symptoms and dropout rates. Additionally, and taking into account clinician-perceived barriers to PE in terms of serious adverse events and symptom exacerbation (van Minnen, Hendriks, & Olff, 2010), we also evaluated treatment safety. Finally, to examine which patients might benefit most from this iPE programme, we aimed to identify distinct treatment response trajectories and explored predictors of these response patterns.

## 2. Method

### 2.1. Participants

**Figure 1.**
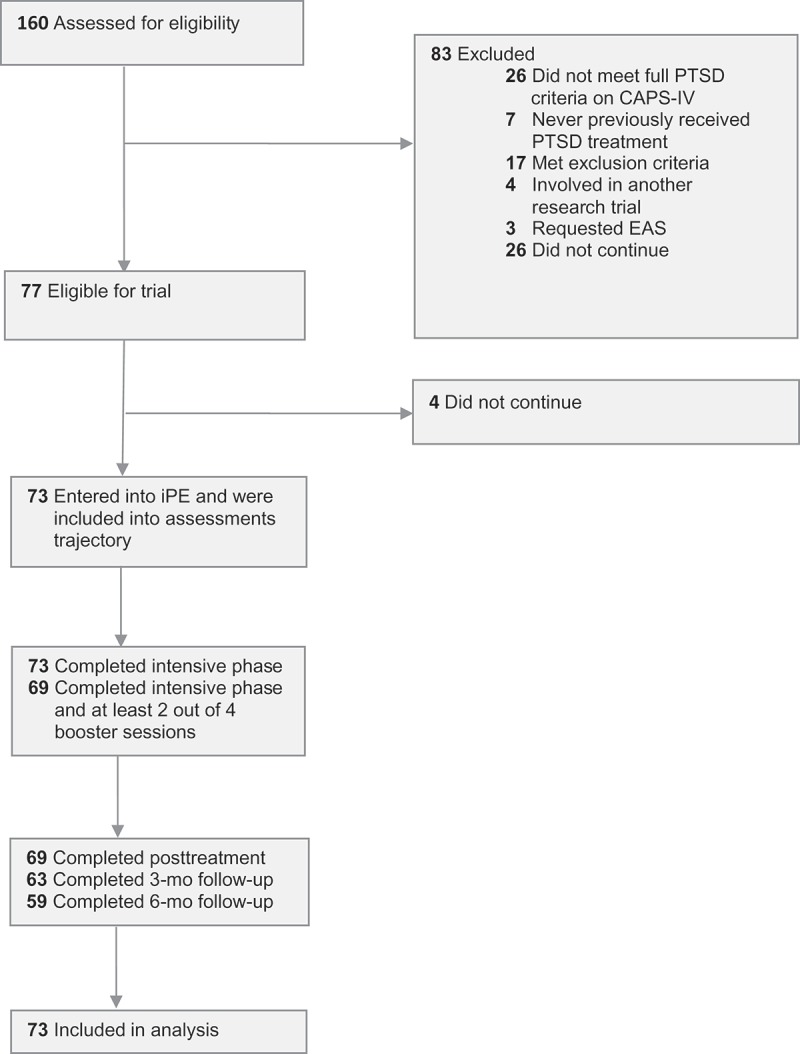
Flow diagram of patient recruitment and trial progress. Note: CAPS-IV = Clinician-Administered PTSD Scale; EAS = euthanasia or assisted suicide; iPE = intensive prolonged exposure; mo = month; PTSD = posttraumatic stress disorder.

Participants (*N *= 73) were regular referrals to a Dutch outpatient mental health clinic specialized in the treatment of anxiety disorders (Figure 1). Inclusion criteria were: (a) age ≥ 18 years; (b) history of multiple interpersonal traumas (repeated sexual abuse and/or repeated physical abuse); (c) meeting full DSM-IV-TR (APA, 2000) diagnostic criteria for PTSD established with the Clinician-Administered PTSD Scale (CAPS-IV; Blake et al., 1995); and (d) history of multiple treatment attempts. Exclusion criteria were: (a) suicide attempt within eight weeks prior to study entry (i.e. suicidality without imminent threat was not an exclusion criterion); (b) inability to speak and write Dutch; (c) severe intellectual impairment defined as an estimated IQ of 70 or less; and (d) comorbid medical conditions requiring more immediate care. Participants were allowed to take psychotropic medication; those on medication (76.7%) were asked to remain on a stable dosage.

### 2.2. Treatment

The iPE therapy programme (see Hendriks, de Kleine, van Rees, Bult, & van Minnen, 2010) was based on Foa’s PE protocol (Foa, Hembree, & Rothbaum, 2007) with the difference that, instead of weekly sessions, the intervention was delivered in a highly intensive format. Treatment started with a 90-minute session aimed at psycho-education and case conceptualization, i.e. establishing a hierarchy of the four most relevant traumatic experiences. The intensive phase consisted of four treatment days delivered within one week. On each of the four treatment days, the participants received three daily individual 90-minute sessions (4.5 hours of treatment per day). Every day, session 1 comprised prolonged imaginal exposure during which the participant was asked to recount aloud the traumatic memory as detailed and vividly as possible, with closed eyes and in the present tense. Session 2 comprised exposure with the participant being instructed to draw the scene(s) of the hotspots of the traumatic memory, each scene on a separate sheet of paper, including all anxiety-provoking details of the hotspots in the drawing (similarly to the instructions during the imaginal exposure). Session 3 included exposure in vivo to trauma-related situations and material. During this intensive phase, patients did not receive any homework assignments because of time constraints. After the intensive phase, participants received four weekly 90-minute PE booster sessions in combination with homework assignments (booster phase) to promote a sound translation and generalization of treatment outcomes to other contexts and everyday life. These booster sessions each comprised prolonged imaginal exposure and exposure in vivo, with homework assignments consisting of listening to the audiotaped imaginal exposure of the previous booster session, drawing the scene(s) of the hotspots of the same traumatic memory, and continuing exposure in vivo as practiced during the previous booster session, all on a daily basis.

### 2.3. Procedure

This study was an open clinical trial and recruitment took place between 2012 and 2015 (registered at trialregister.nl; NTR5931). Patients completed a screening with a therapist who assessed trauma characteristics, PTSD diagnosis using the CAPS-IV (Blake et al., 1995), psychiatric treatment history, and suicidality using the MINI (Sheehan et al., 1998), to determine the inclusion and exclusion criteria. The DSM-IV Axis I diagnoses of any comorbid disorders were also assessed with the MINI (Sheehan et al., 1998). Eligible patients were invited to participate in the study. Participants signed informed consent and completed the baseline assessment.

### 2.4. Measures

The participants’ demographic characteristics were recorded at baseline. Clinician-administered PTSD diagnostic status and symptom severity (past week version), as well as self-reported PTSD symptom severity, were evaluated at baseline, posttreatment (one week after the last booster session), and at three- and six-month follow-up. In addition, self-reported PTSD symptom severity was assessed at each booster session.

*Primary Outcome*. Trained independent research assistants (BSc or MSc in Psychology) interviewed participants using the CAPS-IV (Blake et al., 1995), a clinician-rated structured interview developed to test for symptom severity as well as the presence of a PTSD diagnosis. The CAPS-IV has excellent internal consistency (Cronbach’s α = .94) and interrater diagnostic agreement (Blake et al., 1995). Interrater reliability for a PTSD diagnosis was *k* = 0.92, and *r* = 0.99 for the total severity score in our study (based on 10% randomly selected interviews). Self-reported PTSD severity was monitored using the Dutch translation of the PTSD Symptom Scale, Self-Report (PSS-SR; Foa, Riggs, Dancu, & Rothbaum, 1993), a 17-item questionnaire to rate the frequency of PTSD symptoms. The internal consistency has been shown to be high (Cronbach’s α = .91; Foa et al., 1993) and the Dutch version has also shown to have good internal consistency (Mol et al., 2005).

*Dropout, Adversities and Symptom Exacerbation*. Dropout was recorded by the therapist; treatment completion was defined as having completed the intensive phase of the treatment (four weekdays) and at least two of the four booster sessions. Serious adverse events defined as any medical occurrence that results in death, is life-threatening, requires inpatient hospitalization, results in persistent or significant disability/incapacity, or requires intervention to prevent permanent impairment, were reported. Furthermore, participants reported suicidal ideation, self-harm, aggressive behaviour, and the sense of losing control on an 11-point Likert scale ranging from ‘no, not at all’ (0) to ‘yes, very much’ (10), at baseline and at the end of the intensive phase. Symptom exacerbation was assessed by calculating PSS-SR difference scores from baseline to booster 1 (deterioration during intensive phase), from baseline to posttreatment (deterioration during the treatment including the booster phase), and from baseline to the six-month follow-up. Symptom exacerbation was defined as an increase of 7 or more points on the PSS-SR (Doane, Feeny, & Zoellner, 2010).

*Potential Predictors*. Potential predictors of treatment outcome were measured at baseline or early treatment and all variables were treated as continuous variables, unless otherwise indicated. Potential predictors were assigned to one of three domains: (1) the demographic domain: age, educational level (primary, secondary, vocational, higher vocational education, or university), and living condition (as a categorical variable: living alone vs. together with partner or other people like children or parents); (2) the clinical domain: PTSD symptom severity as assessed using the PSS-SR (Foa et al., 1993), depressive symptom severity as measured with the Beck Depression Inventory (BDI-II; Beck, Ward, Mendelson, Mock, & Erbaugh, 1961), dissociative symptom severity as assessed using the Dissociative Experiences Scale (DES; Bernstein & Putnam, 1986), current severity of borderline personality disorder manifestations as measured with the Borderline Personality Disorder symptom checklist (BPD-47 symptom checklist; Arntz et al., 2003), and psychoactive medication use (as a categorical variable: yes, no); and (3) the fear habituation domain: fear activation during the first exposure session, calculated as the highest given Subjective Units of Distress (SUD) rating (SUD peak) on a 0–10 point scale (no anxiety to maximum anxiety), within-session fear habituation during the first session, calculated as SUD peak minus the latest SUD rating at the end of the first exposure session, and between-session fear habituation, calculated as the difference between SUD peak scores from the first and second imaginal exposure session (Rauch, Foa, Furr, & Filip, 2004; van Minnen & Hagenaars, 2002).

### 2.5. Therapist training and treatment fidelity

All participants were treated by a team of qualified clinicians holding a master’s degree in clinical psychology who were trained in PE therapy for PTSD; all participated in twice-weekly group supervision sessions with a senior therapist (AVM). Therapist adherence to the treatment protocol was verified during the supervision sessions and all deviations from the treatment protocol during the intensive phase were documented and reported by the therapists after each session. The treatment protocol had been violated (e.g. no complete exposure during a session) in 2.1% of all treatment sessions (*N* = 876).

### 2.6. Data analysis

*Treatment Effect*. A mixed models procedure for repeated measures analysis was conducted with statistical software (SPSS 22; IBM SPSS) to analyse scores on the CAPS-IV and PSS-SR with time (baseline, posttreatment, three- and six-month follow-up) as the main fixed effect and an unstructured covariance matrix for the repeated factor time. Effect sizes were calculated for all analyses and interpreted using Cohen’s (1988) criteria. Response on the CAPS-IV (decrease from baseline of ≥ 10), loss of diagnosis (response + no longer meeting ‘1/2’ symptom criteria and CAPS-IV severity score < 45), and remission (loss of diagnosis and CAPS-IV severity score < 20) were calculated (Schnurr & Lunney, 2016). Also, response and remission rates on the PSS-SR were computed, where response was defined as a decrease from baseline on the PSS-SR of 7 or more points (Doane et al., 2010). Loss of diagnosis was defined as a PSS-SR severity score ≤ 20, and remission was defined as a PSS-SR severity score ≤ 10 (Cooper et al., 2017).

*Clustering Analysis*. To investigate change patterns during distinct stages of the treatment, classification was based on response at four different time frames to establish: (1) response to the intensive phase, before the first booster session; (2) response during the booster phase as determined by averaging the scores of the four booster sessions to reflect the process during the booster phase; (3) posttreatment response; and (4) response at follow-up as established by averaging the scores of the two follow-up assessments. In accordance, four difference scores of self-reported PTSD were computed: baseline score minus booster 1 score (d1); baseline score minus mean score of booster 1 to 4 (d2); baseline score minus posttreatment score (d3); and baseline score minus the mean score of the three- and six-month follow-ups (d4). To deal with 17 missing values (6.2%) of these difference scores, multiple imputation was applied (Basagaña, Barrera-Gómez, Benet, Antó, & Garcia-Aymerich, 2013) using the R software package developed by Buuren and Groothuis-Oudshoorn (MICE; 2011) to obtain 100 imputed data sets. On these datasets, k-Means cluster analysis (Steinley, 2006) was carried out using the R software package developed by Barrera-Gómez and Basagaña (MICLUST; 2013) to identify clusters of similar patterns of PSS-SR scores. In k-Means clustering an iterative process reallocates participants to a certain number of clusters in order to minimize the within-cluster variance. A squared Euclidean distance metric was calculated to distribute two-way, two-mode data (*N* objects each having measurements on d1 to d4) into clusters such that the distance for any object and the centroid of its respective cluster is at least as small as the distances to the centroids of the remaining clusters. To gain insight into response patterns beyond response or non-response, we allowed the number of clusters to vary between 3 and 6 and identified the optimal number using CritCF as a goodness of fit measure to evaluate the quality of each partition (Breaban & Luchian, 2011). After the final selection of clusters based on CritCF, participants were allocated to the cluster they were assigned to in most of the imputed data sets. As a final step, the clusters were interpreted in terms of average response (decrease from baseline on the PSS-SR of 7 or more points; Doane et al., 2010), loss of diagnosis, and remission (respectively defined as PSS-SR ≤ 20 and PSS-SR < 10; Cooper et al., 2017).

*Prediction Analysis*. Multinomial logistic regression analyses were conducted with statistical software (SPSS 22; IBM SPSS) to investigate associations between potentially predictive variables and cluster (as a nominal dependent variable with more than two categories). Due to the explorative nature of the prediction analysis, we aimed at generating (instead of testing) hypotheses regarding the associations between potential predictive variables and the clusters. In line with previous work exploring predictors of outcome, potential predictors were classified into different domains and a stepwise procedure was used for each predictor domain (de Kleine, Hendriks, Smits, Broekman, & van Minnen, 2014; Fournier et al., 2009). In step 1, a model including all variables for a given domain was tested. The terms that were significant at *p *< .20 were retained in step 2, where these residual variables for this specific domain were tested again. Subsequently, step 3 retained the terms from step 2 that were significant at *p *< .10, and step 4 retained the terms from step 3 that were significant at *p *< .05. Finally, all terms that were significant at *p *< .05 at step 4 (within each domain) were included in a final model (assessing predictors across all domains).

## 3. Results

Figure 1 shows the participant flow through the study. The characteristics of the study population are presented in Table 1. From April 2012 through August 2015, 73 participants completed the baseline assessment and started iPE. All participants had experienced multiple interpersonal trauma, with the majority reporting childhood (at age ≤ 16 years) abuse: 71.2% childhood sexual abuse and 63.0% childhood physical abuse. The participants were characterized by a current medium to high suicide risk, severe posttraumatic, depressive, and dissociative symptoms, and manifestations of borderline personality disorder. All reported symptoms of complex PTSD (WHO ICD-11 criteria; Cloitre, Garvert, Brewin, Bryant, & Maercker, 2013), i.e. affect dysregulation, negative self-concept, and interpersonal problems.

**Table 1.** Characteristics of the study population (*N *= 73).

Characteristics	
Age in years, mean (*SD*), range	35.9 (11.3), 19–63
Sex, *n*	
Male	10
Female	63
Education, *n* (%)	
Primary education	0 (0.0)
Secondary education	18 (24.7)
Vocational education	24 (32.9)
Higher vocational education	25 (34.2)
University	6 (8.2)
Living condition, *n* (%)	
Alone	31 (42.5)
Together	42 (57.5)
Trauma history, *n* (%)	
Childhood, ≤ 16 years	
Multiple sexual abuse	52 (71.2)
Multiple physical abuse	46 (63.0)
Adulthood, > 16 years	
Multiple sexual abuse	31 (42.5)
Multiple physical abuse	42 (57.5)
Time since trauma in years, mean (*SD*), range	28.2 (12.0), 6–59
Current medium or high suicide risk, *n* (%)	33 (48.5) ^a^
CAPS-IV baseline, mean (*SD*), range	85.1 (17.2), 41–128
PSS-SR baseline, mean (*SD*), range	33.3 (6.5), 18–46 ^b^
BDI-II baseline, mean (*SD*), range	30.6 (11.1), 0–51 ^b^
DES baseline, mean (*SD*), range	22.7 (13.3), 0–56 ^c^
DES baseline, score above clinical cut-off ^d^, *n* (%)	20 (32.8) ^c^
BPD-47 baseline, mean (*SD*), range	103.7 (27.2), 51–203 ^b^
BPD-47 subscales baseline, mean (*SD*), range	
Affect dysregulation ^e^	32.3 (9.0), 19–64 ^b^
Negative self-concept ^f^	20.1 (6.0), 8–36 ^b^
Interpersonal problems ^g^	7.4 (3.1), 3–15 ^b^
Axis I and II comorbidity (current), *n* (%)	68 (93.2)
1–2 comorbid disorder	45 (61.6)
≥ 3 comorbid disorders	23 (31.5)
Comorbid depressive disorder, *n* (%)	50 (68.5)
At least one comorbid anxiety disorder, *n* (%)	23 (31.5)
At least one comorbid personality disorder, *n* (%)	35 (47.9)
Receiving psychotropic medication, *n* (%)	56 (76.7)
Duration of PTSD in years, mean (*SD*), range	10.6 (9.7), 1–40

*Note*: BDI-II = Beck Depression Inventory Second Edition; BPD-47 = Borderline Personality Disorder Checklist; CAPS-IV = Clinician-Administered PTSD Scale; DES = Dissociative Experiences Scale; PSS-SR = PTSD Symptom Scale, Self-Report; PTSD = posttraumatic stress disorder.

^a^ *N *= 68

^b^ *N *= 69

^c^ *N *= 61

^d^ Clinical cut-off defined as a DES severity score of 25 (Boon & Draijer, 1993).

^e^ Sum score of the ‘Mood’, ‘Impulsivity’, and ‘Anger’ subscales.

^f^ As measured by the ‘Identity/Self-concept’ subscale.

^g^ As measured by the ‘Relationships’ subscale.

**Figure 2.**
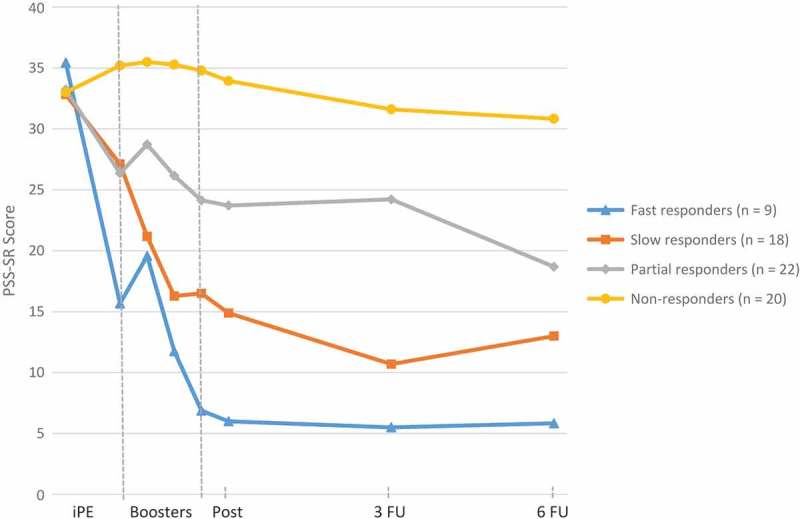
Average PTSD Symptom Scale, Self-Report (PSS-SR) scores before, during, and after treatment within each cluster. Note: iPE = intensive prolonged exposure; Post = Posttreatment; PSS-SR = PTSD Symptom Scale, Self-Report; 3 FU = three-month follow-up; 6 FU = six-month follow-up. Average scores are based on completed assessments.

### 3.1. Treatment effects

The mixed model analysis revealed a main effect of time on both the CAPS-IV (*F*(3,63) = 38.58, *p* < .001) and the PSS-SR scores (*F*(3,60) = 35.84, *p* < .001). There was a decrease in CAPS-IV and PSS-SR scores between baseline and posttreatment that persisted during the three- and six-month follow-ups with large effect sizes (Table 2), reflecting an improvement of clinically observed as well as self-reported PTSD symptoms.

**Table 2.** Primary outcomes at baseline, posttreatment, three- and six-month follow-up ^a.^

Outcome	Baseline	Posttreatment	Three-month follow-up	Six-month follow-up
CAPS-IV, mean (*SD*)	85.1 (17.2)	55.0 (30.8)	50.9 (32.9)	49.6 (30.9)
Effect size	NA	1.21	1.30 ^b^	1.42 ^b^
PSS-SR, mean (*SD*)	33.3 (6.5)	21.7 (11.6)	21.1 (12.5)	20.3 (12.7)
Effect size	NA	1.23	1.23 ^b^	1.29 ^b^

Note: CAPS-IV = Clinician-Administered PTSD Scale; PSS-SR = PTSD Symptom Scale, Self-Report.

^a^ All outcomes reflect the estimated marginal mean from the mixed model analysis. Effect sizes are Cohen’s *d* based on estimated data from the mixed model analysis.

^b^ Effect sizes are based on follow-up compared to baseline scores.

Response and remission rates with regard to PTSD symptoms are listed in Table 3. Fifty-two participants (71.2%) showed posttreatment response, 22 (30.1%) a loss of diagnosis, and 10 (13.7%) achieved posttreatment remission according to the CAPS-IV. A chi-square goodness-of-fit test indicated that both loss of diagnosis and remission percentages had further improved:  loss of diagnosis at the six-month follow-up χ*^2^*(1) = 7.92, *p* = .005, and remission at the three- and six-month follow-ups, χ*^2^*(1) = 5.68, *p* = .017. Based on self-reported PTSD symptoms (PSS-SR), 43 participants (58.9%) showed posttreatment response, 29 (39.7%) a loss of diagnosis, and 10 (13.7%) having achieved posttreatment remission. A chi-square goodness-of-fit test indicated that PSS-SR remission percentages had further improved at three months (χ*^2^*(1) = 4.17, *p* = .041) and at six months (χ*^2^*(1) = 5.68, *p* = .017).

**Table 3.** Observed response, loss of diagnosis, and remission rates according to the CAPS-IV and PSS-SR.

Outcome	Posttreatment	Three-month follow-up	Six-month follow-up
CAPS-IV ^a^			
Response, *n* (%)	52 (71.2)	54 (74.0)	58 (79.5)
Loss of diagnosis, *n* (%)	22 (30.1)	29 (39.7)	33 (45.2)
Remission, *n* (%)	10 (13.7)	17 (23.3)	17 (23.3)
PSS-SR ^b^			
Response, *n* (%)	43 (58.9)	44 (60.3)	47 (64.4)
Loss of diagnosis, *n* (%)	29 (39.7)	31 (42.5)	34 (46.6)
Remission, *n* (%)	10 (13.7)	16 (21.9)	17 (23.3)

Note: CAPS-IV = Clinician-Administered PTSD Scale; PSS-SR = PTSD Symptom Scale, Self-Report.

^a^ Response defined as decrease from baseline ≥ 10 points; loss of diagnosis defined as response plus no longer meeting ‘1/2’ symptom criteria plus a severity score < 45; remission defined as loss of diagnosis plus a severity score < 20 (Schnurr & Lunney, 2016).

^b^ Response defined as a decrease from baseline ≥ 7 points (Doane et al., 2010); loss of diagnosis defined as a severity score ≤ 20; remission defined as a severity score ≤ 10 (Cooper et al., 2017).

### 3.2. Dropout, adversities, and symptom exacerbation

None of the participants dropped out during the intensive phase and 95% completed the treatment as defined as the intensive phase and at least two booster sessions. During the iPE, a single serious adverse event, a psychiatric hospitalization aimed at suicide prevention, occurred. Paired-samples *t*-tests showed no differences from baseline to the end of the intensive phase in self-reported suicidal ideation (baseline *M* = 2.3, *SD* = 2.8; post *M *= 2.2, *SD = *3.1), self-harm (baseline *M* = .8, *SD* = 2.0; post *M *= .9, *SD = *2.2), or aggressive behaviour (baseline *M* = .2, *SD* = .9; post *M *= .2, *SD = *1.1). Sense of losing control decreased from baseline (*M* = 4.5, *SD* = 3.0) to the end of the intensive phase (*M *= 2.7, *SD = *2.8), with the difference, 1.73, BCa 95% CI [.97, 2.50] being significant *t*(66) = 4.52, *p *< .001 and representing a medium effect size, *d = *0.59.

Two participants (2.7%) reported symptom exacerbation on the PSS-SR from baseline to booster 1 (with one participant showing an increase of 7 and the other an increase of 10 points), a third participant from baseline to posttreatment (1.4%, increase of 17 points), while a fourth participant reported symptom exacerbation from baseline to six-month follow-up (1.4%, increase of 8 points).

### 3.3. Clustering analysis

Due to missing baseline scores on the PSS-SR, it was not possible to calculate difference scores for four participants who were, therefore, excluded from the cluster analyses. Use of the CritCF criterion resulted in four clusters: *Fast responders* (*n *= 9), *Slow responders* (*n *= 18), *Partial responders (n *= 22), *Non-responders* (*n *= 20). The *Fast responders* cluster consisted of participants showing self-reported response and a loss of diagnosis immediately after the intensive phase, i.e. before starting the booster phase (average decrease of 19.8 on the PSS-SR, PSS-SR *M_before booster phase_ *= 15.7), who additionally showed a second PSS-SR decrease during the booster phase into remission (average decrease of 29.4 on the PSS-SR from baseline; PSS-SR *M_after booster phase_ *= 6.0). Participants in the *Slow responders* cluster reported no response after the intensive phase (average decrease of 5.7 on the PSS-SR, PSS-SR *M_before booster phase_* = 27.1), but did show a PSS-SR response during the booster phase (average decrease of 17.9 on the PSS-SR from baseline) and a loss of diagnosis (PSS-SR *M_after booster phase_ *= 14.9). Participants classified in the *Partial responders* cluster strictly reported no PSS-SR response during the intensive phase (average decrease of 6.9 on the PSS-SR, PSS-SR *M_before booster phase_* = 26.4). However, their trajectory is characterized by a PSS-SR response during the booster phase (average decrease of 9.5 on the PSS-SR from baseline, PSS-SR *M_after booster phase_* = 23.7) and further decreasing PSS-SR scores during the follow-up period to a loss of diagnosis (PSS-SR *M_6_ _month follow-up_* = 18.7). Finally, participants classified as *Non-responders* had no change in PSS-SR scores at all (average increase of 2.2 on the PSS-SR from baseline to intensive phase, PSS-SR *M_before booster phase_* = 35.2, average increase of 0.9 on the PSS-SR from baseline to post treatment, PSS-SR *M_after booster phase_* = 33.9). Figure 2 shows the longitudinal trajectories of the average PSS-SR scores within each cluster.

### 3.4. Prediction analysis

Supplementary data are available online, providing the results of the stepwise analyses for each of the three domains: demographic characteristics, clinical characteristics, and fear habituation characteristics. Regarding the demographic characteristics, living condition was significantly associated with the *Partial responders* cluster, predicting whether participants belonged to the *Partial responders* cluster or to the *Non-responders* cluster, *b* = −1.86, Wald χ^2^(1) = 7.27, *p* = .007, indicating that participants living alone were more likely to belong to the *Partial responders* cluster than to the *Non-responders* cluster. Within the clinical characteristics domain, no variables were significantly associated with clusters. Results showed that, within the fear habituation characteristics domain, between-session fear habituation was significantly associated with the *Fast responders* cluster. Between-session fear habituation predicted whether participants belonged to the *Fast responders* cluster (*M *= 1.42; 95% CI, -.09–2.92) or the *Non-responders* cluster (*M *= .04; 95% CI, -.53–.61), *b* = .65, Wald χ^2^(1) = 4.06, *p* = .044, indicating that participants showing more between-session fear habituation between the first and second imaginal exposure session were more likely to belong to the *Fast responders* cluster than to the *Non-responders* cluster.

Both significant terms were included in a final model aimed at testing the associations between each variable with cluster while controlling for the other variable (Table 4). Living condition, *b* = −1.89, Wald χ^2^(1) = 7.34, *p* = .007, and between-session fear habituation, *b* = .76, Wald χ^2^(1) = 3.85, *p* = .050, remained significant predictive variables.

**Table 4.** Final model with all significant predictors from the domain models.

		95% CI for Odds Ratio		
Predictor	*b* (SE) ^a^	Lower	Odds Ratio	Upper
*Cluster Fast responders*				
Living condition	1.19 (1.27)	.27	3.29	39.69
Between-session fear habituation	.76 (.39)****	1.00	2.13	4.54
*Cluster Slow responders*				
Living condition	−.65 (.71)	.13	.52	2.09
Between-session fear habituation	−.06 (.25)	.58	.94	1.53
*Cluster Partial responders*				
Living condition	−1.89 (.70)***	.04	.15	.59
Between-session fear habituation	−.22 (.23)	.51	.80	1.26

Note: SE = standard error.

^a^ *b*-values represent unstandardized beta coefficients predicting the chance to belong to one of the specified clusters relative to the *Non-responders* cluster.

* *p *< .20; ** *p *< .10; *** *p *< .05; **** *p *= .05.

## 4. Discussion

Overall, this study suggests, in line with previous RCTs (Ehlers et al., 2014; Zoellner et al., 2017), that PE delivered in an intensive format is effective for PTSD patients. This is especially noteworthy given that our sample mainly consisted of patients who had experienced multiple childhood trauma and reported symptoms of ICD-11 Complex PTSD, expanding the evidence base for the effectiveness of intensive TFT to a more complex patient population. Additionally, all patients had received previous treatments that had proven unsuccessful. Several studies indicate that regular treatment attendance (Tarrier, Sommerfield, Pilgrim, & Faragher, 2000) and a higher frequency of sessions (Gutner et al., 2016), especially early in the treatment, enhances treatment outcome. One possible explanation for these findings is that some PSTD patients have more trouble overcoming their avoidance behaviour and, therefore, are in need of a compact treatment programme instead of weekly standard TFT that leave the patients more room to engage in avoidance behaviour between sessions. However, the results of our trial cannot support this conclusion due to the lack of a control group. Future controlled studies that monitor action readiness to change avoidance before and during iPE and booster sessions are needed.

Arguably, the lack of effect in previous treatments might (also) be associated with the high dropout rates, running up to 52% for conventional PTSD interventions (Imel et al., 2013). In the present study, all participants completed the intensive phase and few patients (5%) left the booster phase prematurely. Again, overcoming avoidance behaviour by delivering TFT treatment within a short time frame may be the key mechanism underlying this results, considering that early dropouts are also assumed to prematurely discontinue treatment to avoid their traumatic memories (Szafranski et al., 2017). Although the low dropout rate in this study might point to the fact that intensive TFT can prevent dropout, we may also have included a select population of patients for whom intensive treatment is acceptable, which limits the generalizability of our results.

Besides investigating the effects of iPE, it is important to address potential risks of massed treatments. A first concern here is that results achieved in a short time might not be maintained in the long run, with relapse as a result. We, however, found that the posttreatment PTSD symptom amelioration persisted up to six months. Second, TFT programmes may evoke serious adverse events or symptom exacerbations (Becker, Zayfert, & Anderson, 2004; van Minnen et al., 2010) and these risks are assumed to be higher in intensive treatment programmes and in more vulnerable PTSD patients with ICD-11 Complex PTSD symptoms such as the present population. We found no evidence for this assumption. Our patients showed no serious adverse events during the iPE in terms of suicide attempts and self-reported PTSD symptom exacerbation was rare (< 3%). Only one serious adverse event occurred in terms of a hospitalization aimed at suicide prevention. The low incidence of serious adverse events and symptom exacerbation contributes to the growing evidence that TFTs are safe (Larsen, Stirman, Smith, & Resick, 2016), also if they are delivered in a massed format.

The effectiveness, low dropout rate, and safety of intensive TFT challenges (some) clinicians’ beliefs that complex PTSD patients need tailored treatment that includes an initial stabilization phase of emotion regulation skills training (Cloitre, 2015; Dorrepaal et al., 2013). The results of the present study provide preliminary evidence that intensive stand-alone TFT (without any preparatory or additional module(s)) and, more specifically, stand-alone intensive exposure, is effective and safe for PTSD patients with a likely diagnosis of ICD-11 Complex PTSD. Our results may then contribute to the identification of commonalities (in this case exposure) in the treatment of PTSD and help us to distinguish the most effective treatment components, enabling us to improve the effectiveness of existing TFT programmes (Schnyder et al., 2015).

Although the response rate we obtained (71%) was comparable to the average rate of 60% found in a systematic review (Loerinc et al., 2015), as well as to the response rates of studies with patient populations comparable to ours based on trauma characteristics (Bohus et al., 2013; Cloitre et al., 2010; Schnurr et al., 2007), not all participants improved to the same degree. Our cluster analyses revealed four distinct trajectories based on change patterns: Fast, Slow, Partial, and Non-responders. Similar distinct response groups were also found by other researchers (Allan et al., 2016; Clapp et al., 2016; Galovski et al., 2016; Stein et al., 2012). We observed that for a small proportion of responders (13%) PTSD symptoms decreased immediately after completing the four days of iPE, i.e. before starting the four-week booster phase in which exposure was expanded to the full variety of contexts (Fast responders). The high dose of PE offered within a short time frame may be the key mechanism underlying the gains in this subgroup. This would be in line with previous research showing that a higher frequency of sessions, especially early in the treatment, enhances treatment outcome (Gutner et al., 2016). Next, we observed that 26% of the responders who had not improved during the intensive phase did show a major decrease in PTSD symptoms during the booster phase (Slow responders). There are several possible explanations for this finding. First, this slow response trajectory may indicate that these patients needed more sessions, which is in line with previous findings showing that some patients need additional sessions to benefit from PE (Foa et al., 2005). Additionally or alternatively, the improvement in this subgroup might not necessarily result from the added exposure but rather from the spacing of the additional exposure sessions during the booster phase. Animal studies (see Fitzgerald et al., 2014) showed that, once initiated in a massed way, extinction learning is boosted when subsequent trials are spaced (Cain et al., 2003). Third, the patients in this cluster may have needed exposure in a variety of contexts such as the home environment. It has been suggested that greater variability in terms of exposure contexts promotes generalization, resulting in better treatment outcomes in anxious patients (Craske, Treanor, Conway, Zbozinek, & Vervliet, 2014). In the third cluster (Partial responders), 32% of the patients showed a partial improvement both during the intensive and the booster phase. This distinct group was also found by others (Galovski et al., 2016; Taylor et al., 2001). It is uncertain whether here full recovery can be achieved with additional therapy. Finally, of all our participants, 29% showed no response to treatment (Non-responders), reporting severe PTSD symptoms at all time points. As other studies recorded a similar non-responders cluster (Allan et al., 2016; Stein et al., 2012), this raises the question whether, for some patients, extinction learning is not feasible (Lissek et al., 2005).

From a clinical perspective, it would be worthwhile if we could determine in advance which patient is likely and which patient is unlikely to respond to iPE. We found that the patients showing more between-session fear habituation between the first and second imaginal exposure session were more likely to belong to the Fast responders cluster than to the Non-responders cluster. This is consistent with previous findings (e.g. Cooper, Clifton, & Feeny, 2017) in which between-session fear habituation, but not within-session fear habituation, was found to be related to treatment outcome. Although replication is needed, our finding does suggest that a lack of habituation to anxiety during the early stage of iPE treatment can be used to detect those patients that are unlikely to benefit from exposure-based treatment. Also, patients living alone were more likely to belong to the Partial-responders cluster than to the Non-responders cluster, which result is in contrast to previous findings (Ehlers et al., 2013; Tarrier et al., 2000; van Minnen et al., 2002). However, it is possible that in our highly affected PTSD population, the presence of a partner helps maintain PTSD-related avoidance behaviour. Interestingly, none of the clinical variables were found to be predictors of treatment response. This is promising as it suggests that even patients with (severe) comorbidity might benefit from intensive TFT programmes like ours. Together, these findings suggest that, contrary to what clinicians generally assume, several baseline demographic and clinical variables do not interfere with treatment and are thus no robust contra-indicators of (intensive) TFT interventions. We rather found that early treatment process variables were better predictors of treatment success.

Because our study lacks a control group and had no randomized design, our findings warrant replication in a randomized controlled design. Additionally, the results of the exploratory cluster and prediction analyses should be interpreted with caution given the low number of participants per cluster. Also, although our participants had a history of multiple treatment attempts, we did not use a standard measure to quantify the degree of treatment resistance per participant (Dunlop, Kaye, Youngner, & Rothbaum, 2014). Another limitation is that we assessed suicidal ideation, self-harm, aggressive behaviour, and a sense of losing control with Likert scales rather than validated measures. Furthermore, we based therapist adherence on self-reports instead of video observation. Lastly, we used the DSM-IV-TR (APA, 2000) diagnostic criteria for PTSD and not the DSM-5 (APA, 2013). However, recent research showed that the CAPS-IV diagnosis closely corresponds to the CAPS-5 diagnosis, with most patients diagnosed according to the DSM-IV criteria also meeting the DSM-5 criteria for PTSD (Weathers et al., 2017). We therefore assume that our results also apply to PTSD populations diagnosed using the DSM-5 criteria.

Showing the effectiveness of intensive TFT for PTSD, Ehlers et al. (2014) emphasized that an intensive TFT is of interest when treatment needs to be conducted within a short period of time or when patients prefer a condensed treatment. Our results expand these implications, suggesting that intensive TFT is a feasible next step in the treatment of chronic PTSD patients with a likely diagnosis of ICD-11 Complex PTSD following multiple interpersonal trauma and a history of multiple treatment attempts. However, also within our intensive TFT programme not all participants improved and future research regarding other next step options is needed.

## 5. Conclusion

Although randomized controlled trials are needed to establish the effectiveness of iPE, this open study suggests that iPE is both effective and safe in PTSD patients having suffered multiple interpersonal childhood trauma and reporting ICD-11 Complex PTSD symptoms. Despite previous treatment attempts being unsuccessful, 71% of the patients showed partial or complete response during iPE, with results being maintained up to six months. Serious adverse events and symptom exacerbation were rare and dropout very low.

## Supplementary Material

Supplementary material
