# Supplementary material for: Intensive prolonged exposure therapy for chronic PTSD patients following multiple trauma and multiple treatment attempts
Source: Eur J Psychotraumatol. 2018 Jan 30;9(1):1425574. doi: 10.1080/20008198.2018.1425574 (PMC5795659; doi:10.1080/20008198.2018.1425574)
Supplement: Supplementary material [file ZEPT_A_1425574_SM1244.docx]

**Supplementary data**

Stepwise analysis of demographic characteristics domain

|  |  | 95% CI for Odds Ratio | | |
| --- | --- | --- | --- | --- |
| Predictor | *b* (SE) ^a^ | Lower | Odds Ratio | Upper |
| *Step 1,2* |  |  |  |  |
| *Cluster Fast responders* |  |  |  |  |
| Age | -.01 (.04) | .92 | .99 | 1.07 |
| Educational level | -.49 (.45) | .26 | .62 | 1.48 |
| Living condition | 1.16 (1.20) | .30 | 3.18 | 33.53 |
| *Cluster Slow responders* |  |  |  |  |
| Age | -.05 (.03)* | .89 | .95 | 1.02 |
| Educational level | -.37 (.37) | .33 | .69 | 1.44 |
| Living condition | -.34 (.74) | .17 | .71 | 3.05 |
| *Cluster Partial responders* |  |  |  |  |
| Age | .01 (.03) | .95 | 1.01 | 1.08 |
| Educational level | -.54 (.39)* | .27 | .59 | 1.25 |
| Living condition | -1.77 (.71)*** | .04 | .17 | .69 |
| *Step 3,4* |  |  |  |  |
| *Cluster Fast responders* |  |  |  |  |
| Living condition | .98 (1.18) | .26 | 2.67 | 26.92 |
| *Cluster Slow responders* |  |  |  |  |
| Living condition | -.65 (.71) | .13 | .52 | 2.10 |
| *Cluster Partial responders* |  |  |  |  |
| Living condition | -1.86 (.70)*** | .04 | .16 | .60 |

*Note.* SE = standard error.

^a)^ *b*-values represent unstandardized beta coefficients predicting the chance to belong to one of the specified clusters relative to the *Non-responders* cluster.

* *p* < .20; ** *p* < .10; *** *p* < .05

Stepwise analysis of clinical characteristics domain

|  |  | 95% CI for Odds Ratio | | |
| --- | --- | --- | --- | --- |
| Predictor | *b* (SE) ^a^ | Lower | Odds Ratio | Upper |
| *Step 1* |  |  |  |  |
| *Cluster Fast responders* |  |  |  |  |
| PSS-SR | .10 (.08) | .94 | 1.11 | 1.30 |
| BDI-II | .04 (.06) | .92 | 1.04 | 1.18 |
| DES | -.05 (.04) | .88 | .95 | 1.04 |
| BPD-47 | -.05 (.03)** | .90 | .95 | 1.00 |
| Psychoactive medication | -2.60 (1.22)*** | .01 | .07 | .82 |
| *Cluster Slow responders* |  |  |  |  |
| PSS-SR | .03 (.06) | .91 | 1.03 | 1.16 |
| BDI-II | -.05 (.04) | .88 | .95 | 1.04 |
| DES | -.05 (.03)* | .89 | .95 | 1.01 |
| BPD-47 | .03 (.02)* | .99 | 1.03 | 1.07 |
| Psychoactive medication | 1.10 (.94) | .48 | 3.01 | 18.94 |
| *Cluster Partial responders* |  |  |  |  |
| PSS-SR | .05 (.06) | .94 | 1.05 | 1.19 |
| BDI-II | -.03 (.04) | .90 | .97 | 1.05 |
| DES | -.03 (.03) | .92 | .98 | 1.03 |
| BPD-47 | -.02 (.02) | .94 | .98 | 1.02 |
| Psychoactive medication | .42 (.91) | .26 | 1.52 | 9.09 |
| *Step 2* |  |  |  |  |
| *Cluster Fast responders* |  |  |  |  |
| DES | -.05 (.04) | .88 | .95 | 1.03 |
| BPD-47 | -.03 (.02)* | .93 | .97 | 1.01 |
| Psychoactive medication | -1.94 (1.00)** | .02 | .14 | 1.02 |
| *Cluster Slow responders* |  |  |  |  |
| DES | -.05 (.03)** | .89 | .95 | 1.01 |
| BPD-47 | .02 (.02) | .99 | 1.02 | 1.05 |
| Psychoactive medication | .75 (.87) | .39 | 2.12 | 11.56 |
| *Cluster Partial responders* |  |  |  |  |
| DES | -.02 (.03) | .93 | .98 | 1.03 |
| BPD-47 | -.03 (.02)* | .94 | .98 | 1.01 |
| Psychoactive medication | .35 (.86) | .27 | 1.42 | 7.62 |
| *Step 3,4* |  |  |  |  |
| *Cluster Fast responders* |  |  |  |  |
| DES | -.07 (.04)** | .86 | .93 | 1.00 |
| Psychoactive medication | -1.45 (.89)* | .04 | .24 | 1.35 |
| *Cluster Slow responders* |  |  |  |  |
| DES | -.03 (.03)* | .92 | .97 | 1.02 |
| Psychoactive medication | .47 (.83) | .32 | 1.59 | 8.05 |
| *Cluster Partial responders* |  |  |  |  |
| DES | -.04 (.03)** | .91 | .96 | 1.01 |
| Psychoactive medication | .68 (.82) | .39 | 1.97 | 9.92 |

*Note.* BDI-II = Beck Depression Inventory Second Edition; BPD-47 = Borderline Personality Disorder Checklist; DES = Dissociative Experiences Scale; PSS-SR = PTSD Symptom Scale, Self-Report; SE = standard error.

^a)^ *b*-values represent unstandardized beta coefficients predicting the chance to belong to one of the specified clusters relative to the *Non-responders* cluster.

* *p* < .20; ** *p* < .10; *** *p* < .05

Stepwise analysis of fear habituation characteristics domain

|  |  | 95% CI for Odds Ratio | | |
| --- | --- | --- | --- | --- |
| Predictor | *b* (SE) ^a^ | Lower | Odds Ratio | Upper |
| *Step 1* |  |  |  |  |
| *Cluster Fast responders* |  |  |  |  |
| SUD peak | 1.63 (1.04)* | .66 | 5.09 | 39.07 |
| Within-session fear habituation | .27 (.22) | .85 | 1.31 | 2.03 |
| Between-session fear habituation | .59 (.44)* | .77 | 1.81 | 4.25 |
| *Cluster Slow responders* |  |  |  |  |
| SUD peak | -.33 (.29) | .41 | .72 | 1.26 |
| Within-session fear habituation | .17 (.16) | .86 | 1.19 | 1.64 |
| Between-session fear habituation | .03 (.30) | .58 | 1.03 | 1.84 |
| *Cluster Partial responders* |  |  |  |  |
| SUD peak | -.13 (.28) | .51 | .88 | 1.54 |
| Within-session fear habituation | .00 (.17) | .73 | 1.00 | 1.39 |
| Between-session fear habituation | -.16 (.31) | .47 | .85 | 1.55 |
| *Step 2* |  |  |  |  |
| *Cluster Fast responders* |  |  |  |  |
| SUD peak | 1.61 (1.02)* | .68 | 4.99 | 36.83 |
| Between-session fear habituation | .82 (.38)*** | 1.08 | 2.28 | 4.81 |
| *Cluster Slow responders* |  |  |  |  |
| SUD peak | -.30 (.28) | .42 | .74 | 1.29 |
| Between-session fear habituation | .11 (.29) | .64 | 1.12 | 1.95 |
| *Cluster Partial responders* |  |  |  |  |
| SUD peak | -.13 (.29) | .50 | .88 | 1.55 |
| Between-session fear habituation | -.15 (.30) | .48 | .86 | 1.54 |
| *Step 3, 4* |  |  |  |  |
| *Cluster Fast responders* |  |  |  |  |
| Between-session fear habituation | .65 (.32)*** | 1.02 | 1.91 | 3.58 |
| *Cluster Slow responders* |  |  |  |  |
| Between-session fear habituation | -.06 (.25) | .58 | .95 | 1.54 |
| *Cluster Partial responders* |  |  |  |  |
| Between-session fear habituation | -.21 (.23) | .52 | .81 | 1.28 |

*Note.* SE = standard error; SUD = subjective units of distress.

^a)^ *b*-values represent unstandardized beta coefficients predicting the chance to belong to one of the specified clusters relative to the *Non-responders* cluster.

* *p* < .20; ** *p* < .10; *** *p* < .05
